# Controllable movement of single-photon source in multifunctional magneto-photonic structures

**DOI:** 10.1038/s41598-020-61811-8

**Published:** 2020-03-16

**Authors:** Thi Huong Au, Amber Perry, Jeff Audibert, Duc Thien Trinh, Danh Bich Do, Stéphanie Buil, Xavier Quélin, Jean-Pierre Hermier, Ngoc Diep Lai

**Affiliations:** 1 Laboratoire Lumière, Matière et Interfaces, FRE 2036, École Normale Supérieure Paris-Saclay, Centrale Supélec, CNRS, Université Paris-Saclay, 4 Avenue des Sciences, 91190 Gif-sur-Yvette, France; 2 Groupe d’étude de la matière condensée, Université Paris-Saclay, UVSQ, CNRS, 45 Avenue des États-Unis, 78035 Versailles, France; 3 Lewis & Clark College, 0615 SW Palatine Hill Rd, Portland, OR 97219 USA; 4 Laboratoire de Photophysique et Photochimie Supramoléculaires et Macromoléculaires, UMR 8531, École Normale Supérieure Paris-Saclay, CNRS, Université Paris-Saclay, 4 Avenue des Sciences, 91190 Gif-sur-Yvette, France; 5 Faculty of Physics, Hanoi National University of Education, 136 Xuan Thuy, Cau Giay, 100000 Hanoi, Vietnam

**Keywords:** Magnetic devices, Quantum dots

## Abstract

Quantum dot (QD) coupling in nanophotonics has been widely studied for various potential applications in quantum technologies. Micro-machining has also attracted substantial research interest due to its capacity to use miniature robotic tools to make precise controlled movements. In this work, we combine fluorescent QDs and magnetic nanoparticles (NPs) to realize multifunctional microrobotic structures and demonstrate the manipulation of a coupled single-photon source (SPS) in 3D space via an external magnetic field. By employing the low one photon absorption (LOPA) direct laser writing (DLW) technique, the fabrication of 2D and 3D magneto-photonic devices containing a single QD is performed on a hybrid material consisting of colloidal CdSe/CdS QDs, magnetite Fe_3_O_4_ NPs, and SU-8 photoresist. Two types of devices, contact-free and in-contact structures, are investigated to demonstrate their magnetic and photoradiative responses. The coupled SPS in the devices is driven by the external magnetic field to perform different movements in a 3D fluidic environment. The optical properties of the single QD in the devices are characterized.

## Introduction

Semiconductor quantum dots (QDs) are potential emitters for applications such as opto-electronic and bio-devices. Due to their high photostability and color size-tunable properties, they are widely used in solar cells^1^, light emitting diodes^2,3^, bio-labeling^4^ and bio-imaging^5^ due to their high photostability and color size-tunable properties. At the single emitter level, semiconductor QDs are a promising non-classical light of room-temperature single-photon source (SPS)^6,7^ and entangled photon source^8^ in quantum applications. Recently, the quantum states have been exploited in various “hybrid” mechanical systems^9–13^ that can be functionalized with an electrode, magnet or mirror. In such applications, it is critical to integrate SPS-single QD into different functionalized structures so that their optical properties can be optimized and/or interfaced with the modulation of external factors. A typical approach involves controlling the electric^14,15^ and optical fields^16,17^. However, the manipulation of the SPS in a three-dimensional (3D) fluid space using a magnetic field has not been previously demonstrated.

In a fluid, precise structures movements are notably harder to control due to additional mechanical degrees of freedom and unbounded displacement of the liquid such as rotational flows, evaporating fluctuations, and free surface with tension force^18–20^. With the aid of an external magnetic field, functionalized structures and devices have recently been controlled to propel and target^21,22^ in the fluid, thus demonstrating multifunctional capabilities. These structures have bright prospects for accessing new quantum mechanical systems, which has motivated our research that is working towards controlling and manipulating the motion of a SPS coupled into mechanical photonic structures.

In this work, we demonstrate for the first time, the motion manipulation of a SPS (colloidal QD) in a 3D fluidic space. Multifunctional devices, each containing a single QD, are fabricated on a hybrid material consisting of colloidal CdSe/CdS QD, iron oxide Fe_3_O_4_ magnetite nanoparticles (MNPs), and a commercial SU-8 photoresist. The single QDs are deterministically coupled into 2D and 3D magneto-photonic structures using the low-one photon absorption (LOPA) direct laser writing (DLW) technique. By applying an external magnetic field, the coupled SPS can be navigated to perform various controlled movements. Furthermore, two types of devices, contact-free and in-contact structures, are investigated for different manipulation purposes. The fluorescence, magnetic and mechanical properties of the structures are characterized to show their multifunctional capabilities and their potential to be used in various applications.

## Results and discussion

In order to fabricate magneto-photonic devices, we first prepare a hybrid material by incorporating colloidal CdSe/CdS nanocrystals and Fe_3_O_4_ MNPs into commercial SU-8 photoresist. The transmission electron microscopy (TEM) images of the QDs and MNPs are shown in Fig. S1(a,b)- Supplementary Information. The colloidal QDs, which have a 2.5-nm-diameter CdSe core and a 5-nm-thick CdS shell, are chemically synthesized as described by Mahler *et al*.^23^. The preparation of the iron oxide MNPs in ethanol (10-nm-diameter NPs) is reported in previous work^24^. By choosing the SU-8 photoresist to host QDs and magnetite NPs, we attempt to synthesize a patternable hybrid composite^25^, allowing for various photo-lithography techniques^26,27^. QDs and MNPs, which are randomly distributed in the SU-8 matrices, are chemically compatible with the photoresist. Within both solutions, cross-linked SU-8 and CdSe/CdS QDs maintain their fluorescence while the MNPs are well-dispersed due to the viscosity effect of the polymer. The concentration of the MNPs has been previously optimized^24,28^ for the particle loading ability of the photoresist, the deformation of the 3D designs, and the magnetic response of the structures. The concentration of the QDs was also tuned to obtain a micrometer interdot-spacing between individual QDs. Note that the coupled QD can be preserved for a long period of time up to several months^25^.

**Figure 1:**
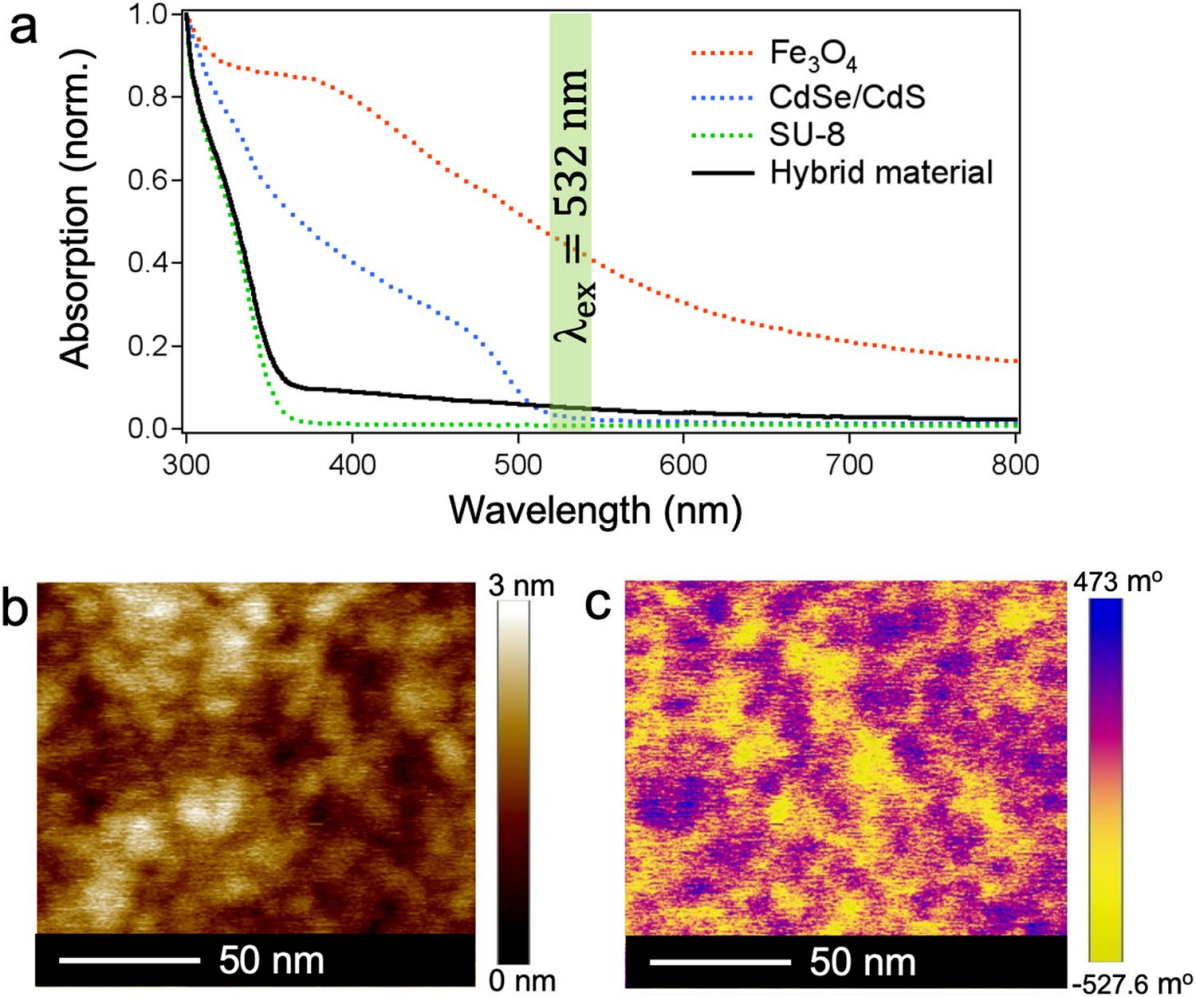
Characterizations of the hybrid material. (**a**) Measured absorption spectra of the hybrid material and separated components at ambient condition. (**b**) Morphological atomic force microscope (AFM) image of a 10-*μ*m-thick film of the hybrid material. (**c**) Magnetic force microscope (MFM) image of the corresponding film.

The absorption spectra of the hybrid material and separated components are measured by a Perkin-Elmer spectrometer and represented in Fig. 1(a). The absorption curve of the hybrid material (black curve) shows a similar trend that of pure SU-8 photoresist (green dashed curve), both of which have a low absorption rate at wavelengths longer than 400 nm and increase sharply at higher energetic wavelengths. Colloidal QDs have strong absorption at wavelengths lower than 500 nm, while MNPs have a typical absorption peak at 400 nm. Due to the low concentration of both QDs and MNPs in the mixture, their contribution to the absorption of the hybrid material is small compared to that of the SU-8. This property is crucial for the fabrication of 2D and 3D structures using the LOPA-based DLW technique on this hybrid material. This is because this technique requires a low absorption rate at the excitation wavelength of 532 nm^29,30^, as indicated by the vertical green line in Fig. 1(a).

Before fabrication, the hybrid material is spin-coated on a glass substrate. Figure 1(b) shows the morphology of a 10-*μ*m-thick film of the hybrid material surface, which has a root-mean-square error (RMS) of 1.5 nm. Note that the NPs doped in the polymer have an unobservable effect on the material due to the nano-molar interspacing of the colloidal QDs and the optimal concentration of the MNPs. The distribution of MNPs is presented in Fig. 1(c) by using a magnetic force microscope (MFM). Individual MNPs near the surface of the material are captured at the stronger spots of magnetic force interaction. The images of a larger scanned area of 100 *μ*m^2^ are shown in Fig. S1(e,f) - Supplementary Information. This provides clear evidence that the MNPs are homogeneously dispersed throughout the hybrid material, and contribute significantly to the high quality of the magneto-photonic devices after fabrication. This is discussed in the following.

**Figure 2:**
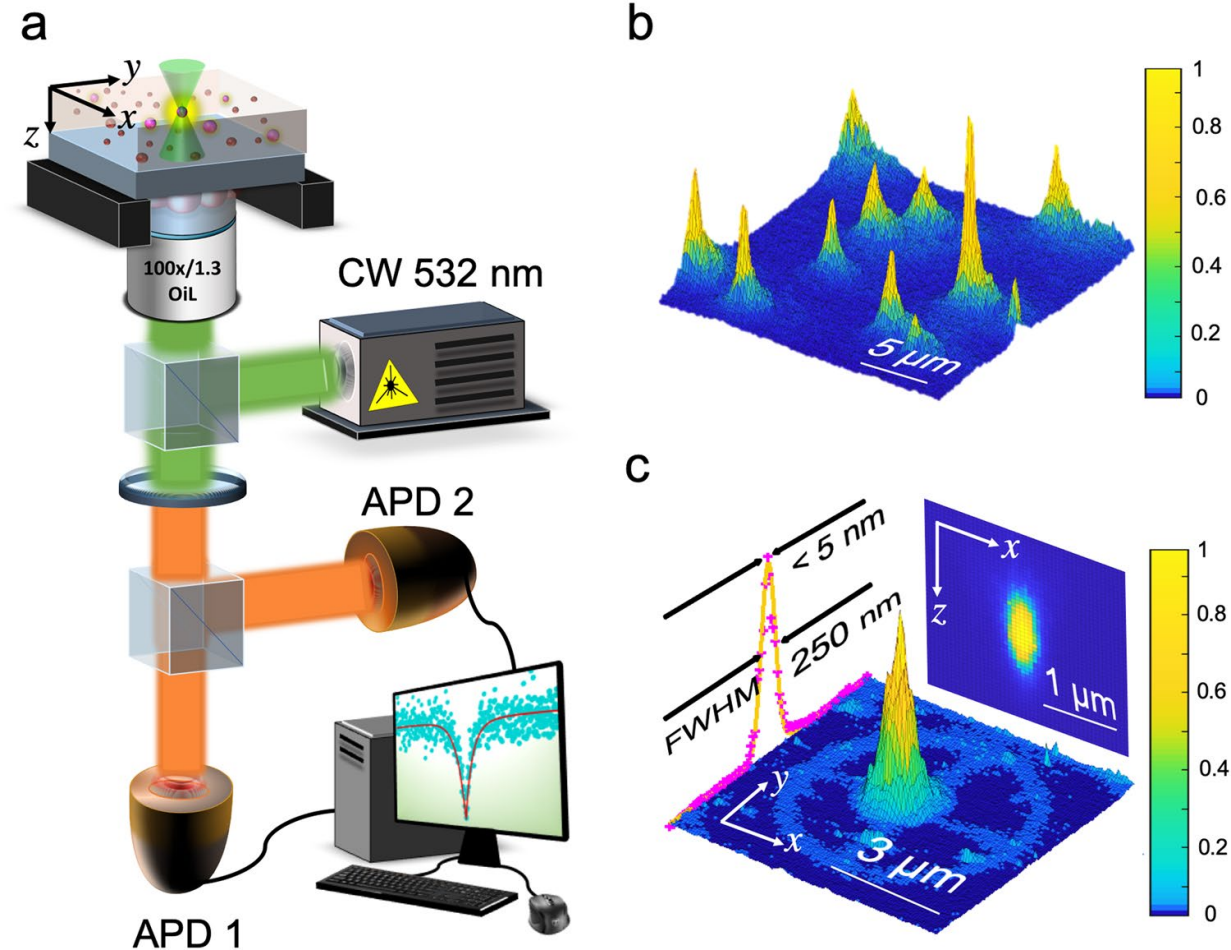
Low-one photon absorption (LOPA) direct laser writing (DLW) technique. (**a**) Schematic of the LOPA-based DLW set-up combined with the Hanbury Brown and Twiss experiment for both fluorescence characterization and structural fabrication. APD: Avalanche Photodiode. Fluorescence map of (**b**) individual QDs within the film of the hybrid material and (**c**) a single QD coupled to a micro-wheel structure after the writing process.

By employing the LOPA-based DLW technique, we can couple single QDs into magneto-photonic structures. Figure 2(a) represents the LOPA set-up. It is combined with an optical confocal configuration used for fluorescent imaging, and with a Hanbury Brown and Twiss (HBT) system used for characterizing the generation of single photons. These tasks are performed using a single 532 mn continuous-wave (CW) laser. The fabrication technique^29^ is based on ultra-low, one-photon absorption of the writing material. This enables the focusing spot to penetrate within the material, thus permitting the ability to fabricate 3D structures. There are two main steps for embedding a single emitter into photonic structures: (i) locate the position of the individual QDs by using a sufficiently low excitation laser power (a few *μ*W) and (ii) fabricate desired structures with a high laser power (a few mW) around the pre-chosen QD. Figure 2(b) presents the location of individual QDs in the hybrid material film. This was obtained by mapping the fluorescence of the QDs with 5 *μ*W laser power. Each bright spot corresponds to a single QD, which is verified by its antibunching property. A single QD is then selected to be coupled into the structure by writing with a 5 mW laser power. A local thermal effect at the focusing spot^31^ causes cross-linked polymeric structures to form immediately; thus there is no need for the conventional post baking step. Figure 2(c) presents the fluorescent image of an embedded QD in a micro-wheel structure after the writing process. The fluorescence of the micro-wheel shape confirms the cross-linking of the SU-8 after the laser scanning. Extracting the data of the QD’s fluorescent spot along one transverse line (in the transverse plane) and one longitudinal line (in the propagation plane) allows one to obtain the peak position. Fitting this with a Gaussian function, the QD position is determined at the peak of the fitted curve with standard error reaching as low as 5 nm and 10 nm for transverse and longitudinal planes, respectively (Fig. 2(c)-subset). Using the same approach, the single QD can be incorporated into any arbitrary structure on demand.

**Figure 3:**
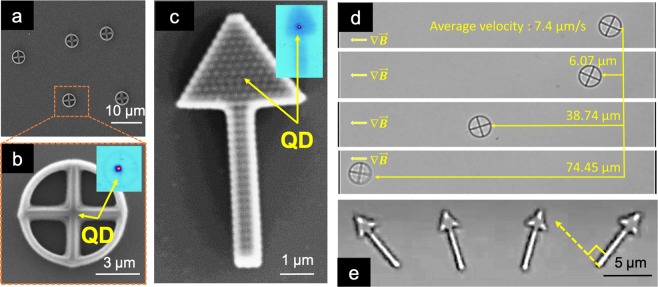
Contact-free devices with incorporated SPS and their movement manipulation in a fluidic environment. Scanning electron microscope (SEM) images of the contact-free structures: (**a**) multiple and (**b**) single micro-wheel structure, (**c**) a micro-arrow structure, with their corresponding fluorescent image in the sub-set. Optical microscope images of (**d**) the micro-wheel performing translational displacement and (**e**) the micro-arrow performing rotational movement.

Since magnetic and mechanical characteristics are shape dependent, the behavior of the hybrid structures can be varied. In order to study this effect, we investigate contact-free magneto-photonic structures. In order to release the devices from the substrate, we polymerized the pattern in the middle of the hybrid material film (see Methods for more details). Figure 3 depicts two designs of the contact-free devices as well as manipulation of their motion. A symmetric micro-wheel is fabricated for this experiment, as shown Fig. 3(a,b). The structure carries a single QD in the center as evidenced by the sub-set fluorescent image. The bright spot indicates the position of the QD, which was characterized to confirm the single photon emission both before and after the fabrication process. After the fabrication of the structures, we demonstrated the translational movement of the structures in a fluidic environment, as shown in Fig. 3(d). Due to the magnetic component preserved within the structures, an external magnetic field generated from a bar magnet can be applied to manipulate them. The magnetic flux density, measured by a digital Gaussmeter (GM07-Hirst Magnetic Instrument, UK) with a transverse probe, is 10 mT at the sample position. The micro-wheel structure was manipulated to perform a linear displacement for distances varying between a few micrometers to hundreds of micrometers. The average velocity of the structure achieved 7.4 *μ*m per second using the above mentioned magnetic flux density. We also present an example of an asymmetric contact free shape in the form of a micro-arrow, as shown in Fig. 3(c). The location of the single QD is indicated by the corresponding fluorescent image. Due to the asymmetric shape, the micro-arrow structure is used to perform rotational movements. Figure 3(e) shows a series of screen-shots of a micro-arrow structure under an optical microscope. In this image, the micro-arrow is submerged in the solution and under the control of a uniform external magnetic field (10 mT), generated from a U-shaped magnet. The resulting movements display rotation at different angles. Comparing the orientation of the applied magnetic field and alignment of the arrow allows us to obtain the linear response of the micro-device. For example, the 90° rotational angle of the magnetic field was completely followed by a 90° rotational angle of the micro-arrow. The responses of the contact-free magneto-photonic devices suggests that by programming the applied external magnetic field, more advanced motion manipulations can be performed on this type of device. This yields much potential in further developments of motion manipulation on the microrobotic platform.

**Figure 4:**
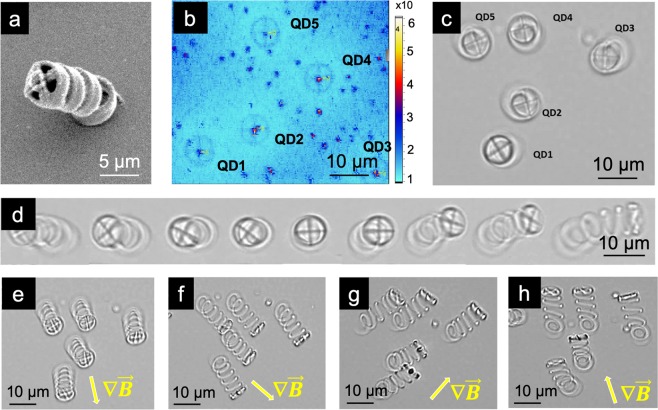
In-contact device and its controllable manipulation. (**a**) Scanning electron microscope (SEM) images of a micro-spring. (**b**) Fluorescent map of fabricated micro-spring structures with a integrated QD at the center of the micro-wheel before the development step. (**c**) The transmission optical microscope image of micro-spring structures corresponding to (**b**) the fluidic environment. (**d**) The transmission optical microscope images of a micro-spring at different bending stage when deliberately changing the direction of the magnetic flux in a semicircular track above the sample. (**e–h**) The orientation of structures toward higher gradient of magnetic field with depicted magnetic field gradient direction.

We also developed a second type of magneto-photonic device by investigating in-contact structures. In this experiment, a single QD is coupled into the center of a micro-wheel that is attached to a vertical micro-spring (a model is illustrated in Fig. S2 - Supplementary Information). As an in-contact structure, micro-springs are fabricated with a fixed position on the glass substrate. This is shown in Fig. 4(a). To incorporate a single QD into the structure, the same approach is used as mentioned above. Figure 4(b) presents the fluorescent map of 5 micro-spring structures with a single QD at the center of each integrated micro-wheel. This fluorescent image was taken before the development process. Thus, the QDs outside of the patterns remain in the SU-8 monomers (uncross-linked area). After the development step, the coupled QDs are preserved within the structures while the others are removed by the development solution. Figure 4(c) shows the transmission microscope image (top view) of the 5 corresponding micro-spring structures. They are stable standing in the fluidic environment when no force is being applied to them.

In the presence of an external magnetic field, these in-contact devices can perform complex movements that display the mechanical properties of the structures in the fluidic environment. By introducing a magnetic field of 10 mT, an attractive interaction of the devices was observed as the structure bent in the direction of higher magnetic flux density. To fully study the linear response of this structure, the direction of the magnetic flux was manipulated in a semicircular track above the sample. The resulting movements of the micro-spring structure are illustrated in a series of screen-shots (Fig. 4(d)). Additionally, we tested a group of 5 micro-spring structures, verifying a uniform response of magnetic patterns in a large area. Figure 4(e–h) presents the different orientations of the group of structures toward the higher magnetic field gradient. The consistent behavior and the linear response of the five individual structures are evident due to the high quality of the hybrid material and the strong magnetic property of the devices.

**Figure 5:**
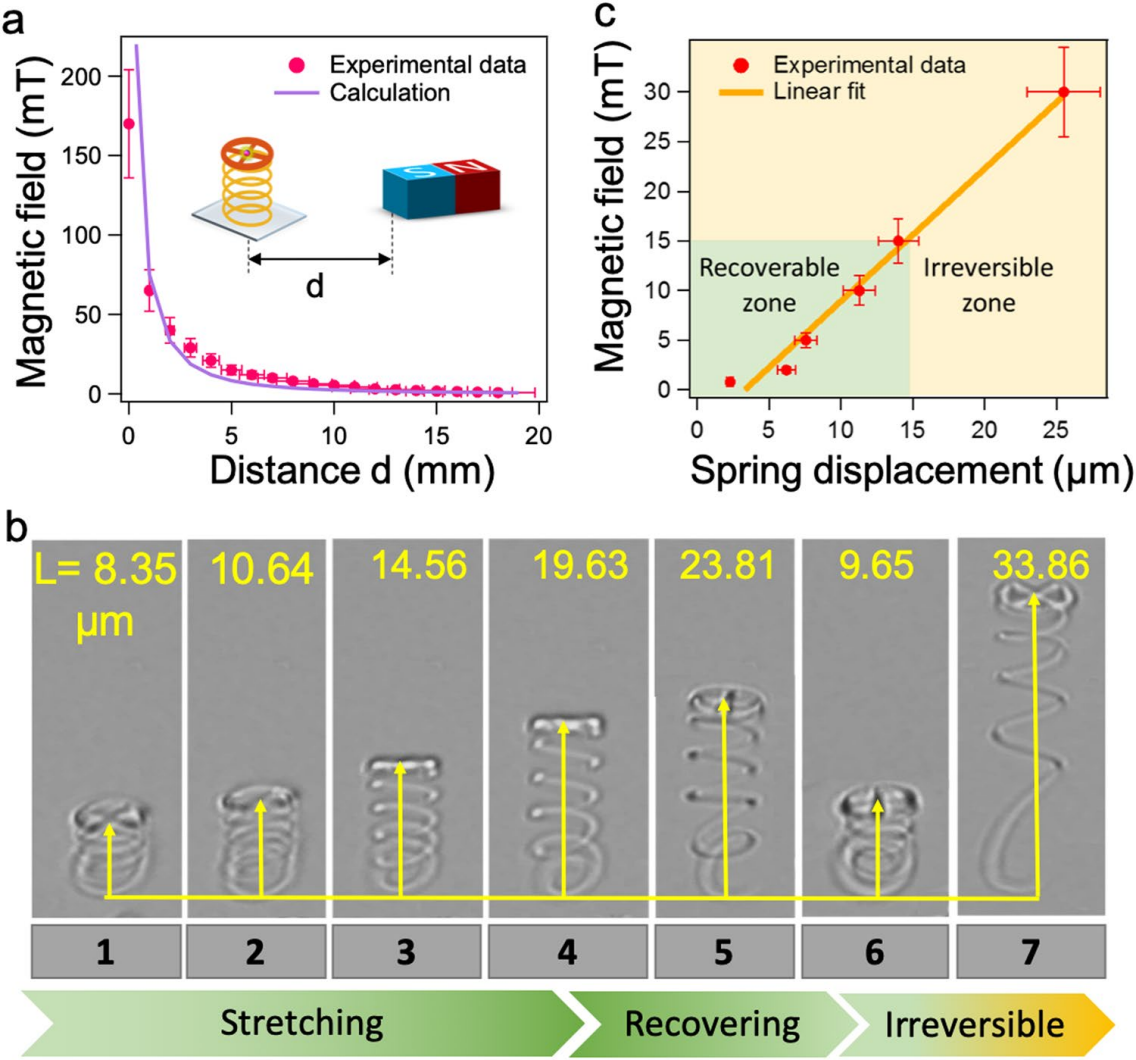
The elasticity of the micro-spring under the influence of an applied external magnetic field. (**a**) The dependence of the magnetic field on the distance, d, from a magnet to the sample position. (**b**) The stretching, recovery, and irreversible performance of a micro-spring structure under the influence of the applied magnetic field, as observed by the transmission microscope. (**c**) The dependence of spring displacement on the applied magnetic field. The green area denotes for the recoverable zone of the micro-spring and the yellow zone denotes for the irreversible zone of the micro-spring.

To provide further insights into the nano-mechanical properties of the hybrid material, we investigated the spring elasticity under the influence of the external magnetic field. Using the same permanent bar magnet equipped with a microscale translator, different magnetic flux densities were generated at the sample position by varying the distance (mm range) between the magnet (one end) and the sample (center position). Figure 5(a) presents the measured magnetic flux density versus the distance (red dots). The maximum detected magnetic flux density (173 mT) was obtained at one end of the magnet and exponentially decreased as we moved the magnet farther from the sample. The measured data is consistent with our calculations (purple line). The attractive force induced from the magnetic field causes the micro-spring structure to stretch. As illustrated in Fig. 5(b), a single micro-spring structure is manipulated to stretch with different extensions, to recover, and to deform in the liquid environment. The extension of the micro-spring (Fig. 5(b)) is measured and graphed in Fig. 5(c) as a function of the applied magnetic field. At an equilibrium state, the micro-spring has a length of around 8.3 *μ*m (Fig. 5(b1)). When the magnetic field increases, the induced magnetic force increases, causing the micro-string to stretch. Note that the micro-spring nearly triples in length at its maximum extension (23.8 *μ*m-Fig. 5(b5)) compared to its length at the equilibrium state. When the magnetic field is removed, the micro-spring returns back to the unstrained position and has a length of 9.65 *μ*m (Fig. 5(b4)). The micro-spring displacement is linearly proportional to the influence of the applied magnetic field, which is proportional to the induced magnetic force. Experimental data is fitted linearly to confirm this behavior of the micro-spring (Fig. 5(c)). When a greater magnetic field (≥15 mT) is applied on the sample, the micro-spring makes an irreversible stretch and becomes deformed (Fig. 5(b7)). All of these characteristics are typical properties of a conventional spring. From these results, it is clear that the fabricated micro-spring structures possess the typical mechanical properties of a spring.

**Figure 6:**
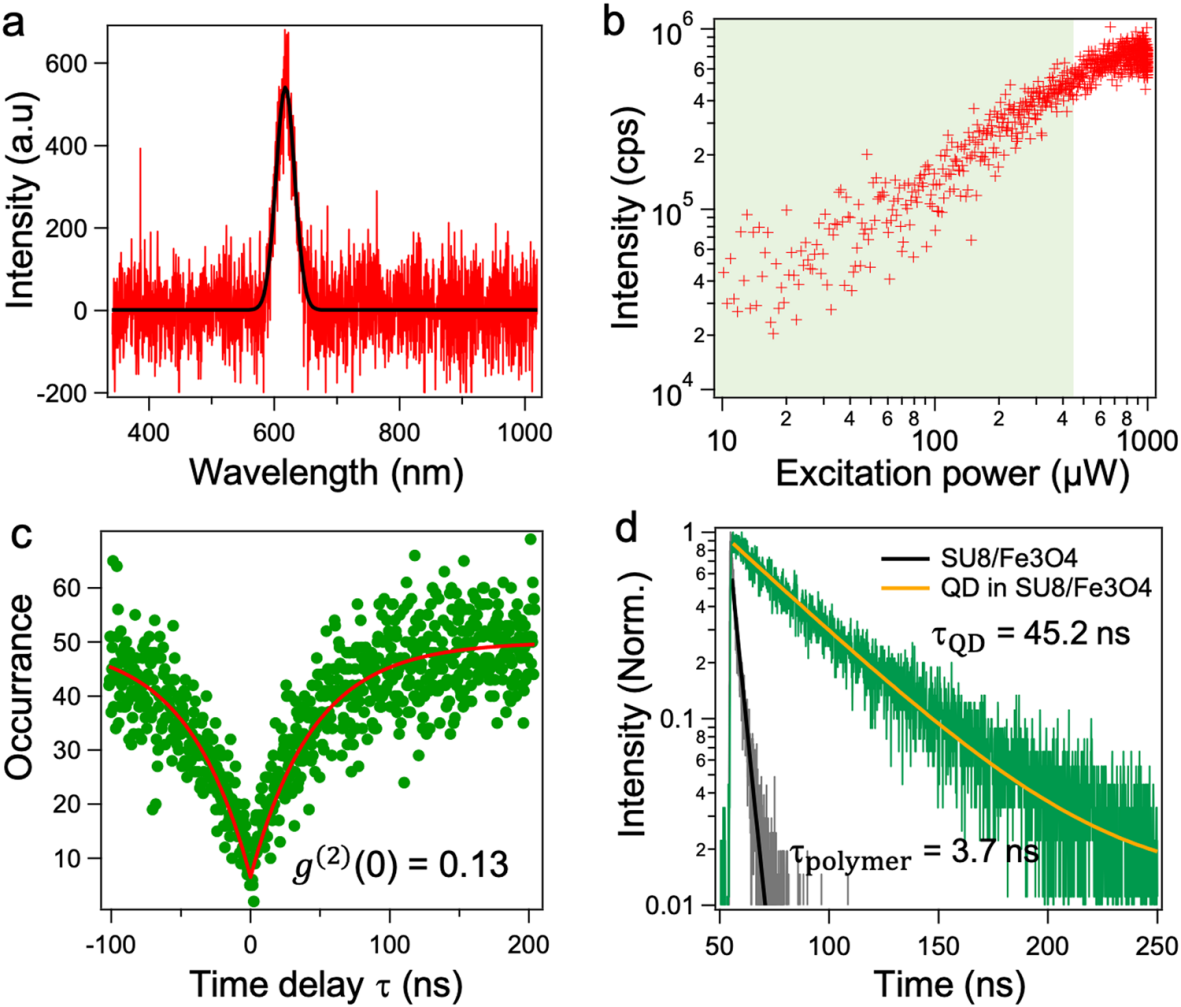
Characterization of the coupled QD after the manipulation. (**a**) Emission spectrum of a coupled QD in the structure fitted with a Gaussian function to extract the emission peak at 620 nm and full-width-half-maximum (FWHM) of around 30 nm. (**b**) The dependence of emission rate of the coupled QD as the function of the excitation power, excited by the green laser at 532 nm. Green area indicates a region obtaining single photon emission *g*^(2)^(0) ≤ 0.5. (**c**) Antibunching curve of the coupled QD with the fitted value of *g*^(2)^(0) ≈ 0.1. (**d**) Time-resolved fluorescence decay of the SU-8/Fe_3_O_4_ mixture and of a QD in the mixture with fitted lifetime of 3.7 ± 0.1 and 45 ± 1 ns, respectively.

After manipulation in the liquid environment, the external magnetic field and the liquid solution are removed to examine the optical properties of the structures. Figure 6(a) presents an emission spectrum of a QD using an Ocean Optic USB2000+ Spectrometer with a resolution of 0.35 nm at room temperature. The curve is fitted with a Gaussian function to extract the emission peak at 620 nm and the full-width-half-maximum (FWHM) of around 30 nm. The emission rate is characterized as the function of the excitation power in the range of 50 *μ*W to 1 mW. The emission rate increases from a thousand counts per second (cps) to around 0.7 million cps and becomes saturated at a power larger than 600 *μ*W (Fig. 6(b)). The antibunching property of the coupled QDs is characterized by the HBT experiment equipped with TimeHarp 260 Correlation card, PicoQuant. The green area in Fig. 6(b) indicates the value of the excitation power and the corresponding fluorescence rate for which we can obtain the single photon emission (*g*^(2)^(0) ≤ 0.5). In order to avoid the over-pumping on QD, resulting in multi-excitation and photo-bleaching, a low excitation power at around 5 *μ*W is commonly used^32^. The single photon quality is obtained at *g*^(2)^(0) ≈ 0.1 with a time bin of 0.4 ns (Fig. 6(c)). The time-resolved fluorescence decays of the SU-8/Fe_3_O_4_ medium and of a QD in this medium are also measured. The exponential fits of these plots indicate lifetimes of 3.7 ± 0.15 and 45.19 ± 0.91 ns, respectively (Fig. 6(d)). These fluorescence behaviors of the coupled QD are consistent with our previous studies^25,33^. This result confirms that no change in the QDs’ optical properties occur after external treatments and manipulations. Due to the chemical-resistant and bio-compatible characteristics of the cross-linked SU-8, the fabricated structures are able to be manipulated in various types of chemical and bio-fluid liquid environments without the degradation of the QD properties.

In conclusion, we have demonstrated for the first time the controllable movement of a SPS within 3D multifunctional magneto-photonic devices. The single QDs have been deterministically coupled into either contact-free or in-contact structures using the LOPA-based DLW technique. Via an external magnetic field, the coupled QDs were manipulated to perform various movements while retaining a bright SPS. The fluorescence, magnetic, and mechanical properties of the devices were then characterized to display a variety of capabilities, thus showing great potential for fluorescence-magnetic bimodal structuring, nano-machining, quantum technology, and other multidisciplinary applications.

## Methods

### Elaboration of the hybrid material

The colloidal CdSe/CdS core/shell QDs were initially diluted in a hexane/octane (C6/C8) solution. The QD solution is changed to toluene in order to disperse them evenly in the SU-8 photoresist (MicroChem.). The QDs in toluene were then gradually injected with a low concentration into a bath of SU-8 monomer with constant stirring. Notice that with a high concentration of QDs one obtains a dispersion of clusters rather than a mono-dispersion of CdSe/CdS QDs in the polymer material. The mixed solution was stirred for 4 hours to evaporate the toluene solution and thus obtain a homogeneous distribution of QDs, resulting in the initial SU-8 viscosity level of the QDs/SU-8 nanocomposite. The iron oxide Fe_3_O_4_ MNPs in ethanol were then added to the mixture followed by a 30-minute treatment in an ultrasonic bath. Different types of epoxy-based negative photoresist, SU-8 2000.5, SU-8 2002, SU-8 2005, SU-8 2010, and SU-8 2025 with different viscosities of 2.49 cSt, 7.5 cSt, 45 cSt, 380 cSt, and 4500 cSt, respectively, were used as candidates for hosting the QDs and MNPs. The dispersion of Fe_3_O_4_ MNPs in the mixture was also investigated with different concentrations ranging from 0.01 to 2 *wt*%. Finally, nanocomposite solutions of various viscosities and concentration levels were stored in a dark, nonmagnetic environment and examined by optical microscopy as a function of time.

### Fabrication of contact-free and in-contact devices

A cw green laser beam (*λ* = 532 nm) is tightly focused into the sample by an oil-immersion (NA = 1.3) objective lens. The sample is translated in 3D space following a controllable trajectory by a high-resolution Piezoelectric Nanopositioning system (P-563 PIMars, PI). The structures are fabricated using different powers (from 10 *μ*W to 40 mW) and different scanning speeds (from 1 *μ*m/s to 8 *μ*m/s). Due to an optically induced thermal effect at the focusing spot, the fabricated structures are completely polymerized (cross-linked) after the scanning (see Fig. S2(a)-Supporting Information) without the post-exposure baking process. Within the structures, the MNPs and QD are preserved in a coupled position. Meanwhile the ones in the SU-8 monomers are removed by the development step. The contact-free structures have a radius of 3 *μ*m, a width of 400 nm for the micro-wheel structure, and a length of 8 *μ*m for the micro-arrow structure.Before releasing the contact-free devices from the glass substrate, a distance from the fabricated structures to the substrate must be considered (about few *μ*m). The monomers layer under the pattern will act as a sacrificial layer which allows one to release the structures into the solution. In contrast with contact-free structures, the in-contact structures were fabricated from the interface of the polymer and the substrate so that the structures are fixed on the glass substrate and thus unable to be manipulated externally. The design dimensions of the in-contact device have a radius R, micro-wheel linewidth *W*_*wheel*_, nano-coil linewidth *W*_*coil*_, vertical pitch P, length of spring L, and number of turns, N, which are 3 *μ*m, 1 *μ*m, 0.5 *μ*m, 1.5 *μ*m, 7 *μ*m and 4, respectively. (see Fig. S2(b) - Supplementary Information). To develop the structures, the samples were emerged in SU-8 developer, then in isopropannol, and finally in distilled water for 2 minutes to get rid of unexposed parts and leaving the desired structures on the glass substrate.

### Manipulation of contact-free and in-contact devices

After the structural writing processes, the devices with a single coupled QD were transferred to a transmission optical microscope for observation and magnetic manipulation. Both contact-free and in-contact devices can be fabricated and manipulated at the same time as illustrated in Fig. S2(c)-Supporting Information. For simplicity, we directly employed the SU-8 developer as a fluidic environment to perform the controlled movement of the devices. After emerging the sample in SU-8 developer, the devices are immediately released into the solution. Next, a permanent bar magnet is introduced to generate an external magnetic field for the movement manipulation (see Fig. S2(d)-Supporting Information). By adjusting the distance from the magnet to the sample we are able to control the magnetic flux density which has different effects on the structures. Consequently, the devices are manipulated and recorded on the optical microscope.

## Supplementary information

Supplementary Information.

## References

[1] Robel I, Subramanian V, Kuno M, Kamat PV (2006). Quantum dot solar cells. harvesting light energy with CdSe nanocrystals molecularly linked to mesoscopic TiO2 films. Journal of the American Chemical Society.

[2] Choi MK, Yang J, Hyeon T, Kim D-H (2018). Flexible quantum dot light-emitting diodes for next-generation displays. npj Flexible Electronics.

[3] Sun B, Marx E, Greenham NC (2003). Photovoltaic Devices Using Blends of Branched CdSe Nanoparticles and Conjugated Polymers. Nano Letters.

[4] Parak WJ, Pellegrino T, Plank C (2005). Labelling of cells with quantum dots. Nanotechnology.

[5] Damalakiene L (2016). Fluorescence-Lifetime Imaging Microscopy for Visualization of Quantum Dots’ Endocytic Pathway. International Journal of Molecular Sciences.

[6] Hoang TB, Akselrod GM, Mikkelsen MH (2016). Ultrafast Room-Temperature Single Photon Emission from Quantum Dots Coupled to Plasmonic Nanocavities. Nano Letters.

[7] Pisanello F (2013). Non-Blinking Single-Photon Generation with Anisotropic Colloidal Nanocrystals: Towards Room-Temperature, Efficient, Colloidal Quantum Sources. Advanced Materials.

[8] Chen GY, Lambert L, Chou CH, Chen YN, Nori F (2011). Surface plasmons in a metal nanowire coupled to colloidal quantum dots: Scattering properties and quantum entanglement. Physical Review B.

[9] Rabl P (2010). A quantum spin transducer based on nanoelectromechanical resonator arrays. Nature Physics.

[10] Stannigel K, Rabl P, Sorensen AS, Zoller. P, Lukin MD (2010). Opto-mechanical transducers for long-distance quantum communication. Physic Review Letters.

[11] Akram U, Kiesel N, Aspelmeyer M, Milburn GJ (2010). Single-photon opto-mechanics in the strong coupling regime. New Journal of Physics.

[12] Chan J (2011). Laser cooling of a nanomechanical oscillator into its quantum ground state. Nature.

[13] O’Connell AD (2010). Quantum ground state and single-phonon control of a mechanical resonator. Nature.

[14] Lin X (2017). Electrically-driven single-photon sources based on colloidal quantum dots with near-optimal antibunching at room temperature. Nature Communications.

[15] Nowak AK (2014). Deterministic and electrically tunable bright single-photon source. Nature Communications.

[16] Garcia S, Maxein D, Hohmann L, Reichel J, Long R (2013). Fiber-pigtailed optical tweezer for single-atom trapping and single-photon generation. Applied Physics Letters.

[17] Yoshie T (2004). Vacuum Rabi splitting with a single quantum dot in a photonic crystal nanocavity. Nature.

[18] Shkarin AB (2019). Quantum optomechanics in a liquid. Physical Review Letters.

[19] Gil-Santos E (2015). High-frequency nano-optomechanical disk resonators in liquids. Nature Nanotechnology.

[20] Paul MR, Cross MC (2004). Stochastic dynamics of nanoscale mechanical oscillators immersed in a viscous fluid. Physical Review Letters.

[21] Peters C (2014). Superparamagnetic Twist-Type Actuators with Shape-Independent Magnetic Properties and Surface Functionalization for Advanced Biomedical Applications. Advanced Functional Materials.

[22] Kim S (2013). Fabrication and Characterization of Magnetic Microrobots for Three-Dimensional Cell Culture and Targeted Transportation. Advanced Materials.

[23] Mahler B (2008). Towards non-blinking colloidal quantum dots. Nature Materials.

[24] Au TH (2017). Direct Laser Writing of Magneto-Photonic Sub-Microstructures for Prospective Applications in Biomedical Engineering. Nanomaterials.

[25] Au TH, Buil S, Quélin X, Hermier J-P, Lai ND (2019). Photostability and long-term preservation of a colloidal semiconductor-based single photon emitter in polymeric photonic structures. Nanoscale Advances.

[26] Au TH, Buil S, Quelin X, Hermier J-P, Lai ND (2019). High directional radiation of single photon emission in dielectric antenna. ACS Photonics.

[27] Perry A (2019). Mask lithography of 2d fluorescent magneto-photonic microstructures for biomedical and quantum applications. SPIE Proc. Colloidal Nanoparticles for Biomedical Applications XIV.

[28] Au TH (2018). Free-floating magnetic microstructures by mask photolithography. Physica B: Condensed Matter.

[29] Do MT (2013). Submicrometer 3D structures fabrication enabled by one-photon absorption direct laser writing. Optics Express.

[30] Mao, F. *et al*. LOPA-based direct laser writing of multi-dimensional and multi-functional photonic submicrostructures. **SPIE Proc. Advanced Fabrication Technologies for Micro/Nano Optics and Photonics X****10115**, 1011509 (2017).

[31] Nguyen DTT, Tong QC, Ledoux-Rak I, Lai ND (2016). One-step fabrication of submicrostructures by low one-photon absorption direct laser writing technique with local thermal effect. Journal of Applied Physics.

[32] Au TH, Buil S, Quélin X, Hermier J-P, Lai ND (2018). Suppression of grey state and optimization of the single photon emission of a colloidal semiconductor at room temperature. Applied Physics Letters.

[33] Au TH, Buil S, Quélin X, Hermier J-P, Lai ND (2018). Coupling of a single photon source based on a colloidal semiconductor nanocrystal into polymer-based photonic structures. Proc. SPIE 10672, Nanophotonics VII.

