# Supplementary material for: Controllable movement of single-photon source in multifunctional magneto-photonic structures
Source: Sci Rep. 2020 Mar 16;10:4843. doi: 10.1038/s41598-020-61811-8 (PMC7075966; doi:10.1038/s41598-020-61811-8)
Supplement: Supplementary file 1 — Supplementary Information. [file 41598_2020_61811_MOESM1_ESM.pdf]

Controllable movement of single photon source in multifunctional magneto-photonic structures

Thi Huong Au[1,2], Amber Perry[1,3], Jeff Audibert[4], Duc Thien Trinh[5], Danh Bich Do[5], Stéphanie Buil[2], Xavier Quélin[2], Jean-Pierre Hermier[2,*], and Ngoc Diep Lai[1,*]

[1]Laboratoire Lumière, Matière et Interfaces, FRE 2036, École Normale Supérieure Paris-Saclay, Centrale Supélec, CNRS, Université Paris-Saclay, 4 Avenue des Sciences, 91190 Gif-sur-Yvette, France.
[2]Groupe d'Étude de la Matière Condensée, Université de Versailles Saint-Quentin-en-Yvelines, CNRS UMR 8635, Université Paris-Saclay, 45 Avenue des États-Unis, 78035 Versailles Cedex, France.
[3]Lewis & Clark College, 0615 SW Palatine Hill Rd, Portland, OR 97219, USA.
[4]Laboratoire de Photophysique et Photochimie Supramoléculaires et Macromoléculaires, UMR 8531 CNRS, École Normale Supérieure de Cachan, Université Paris-Saclay, 61 avenue du Président Wilson, 94235 Cachan, France.
[5]Faculty of Physics, Hanoi National University of Education, 136 Xuan Thuy, Cau Giay, 100000 Hanoi, Vietnam.
[*]jean-pierre.hermier@uvsq.fr, ngoc-diep.lai@ens-paris-saclay.fr

– SUPPLEMENTARY INFORMATION –

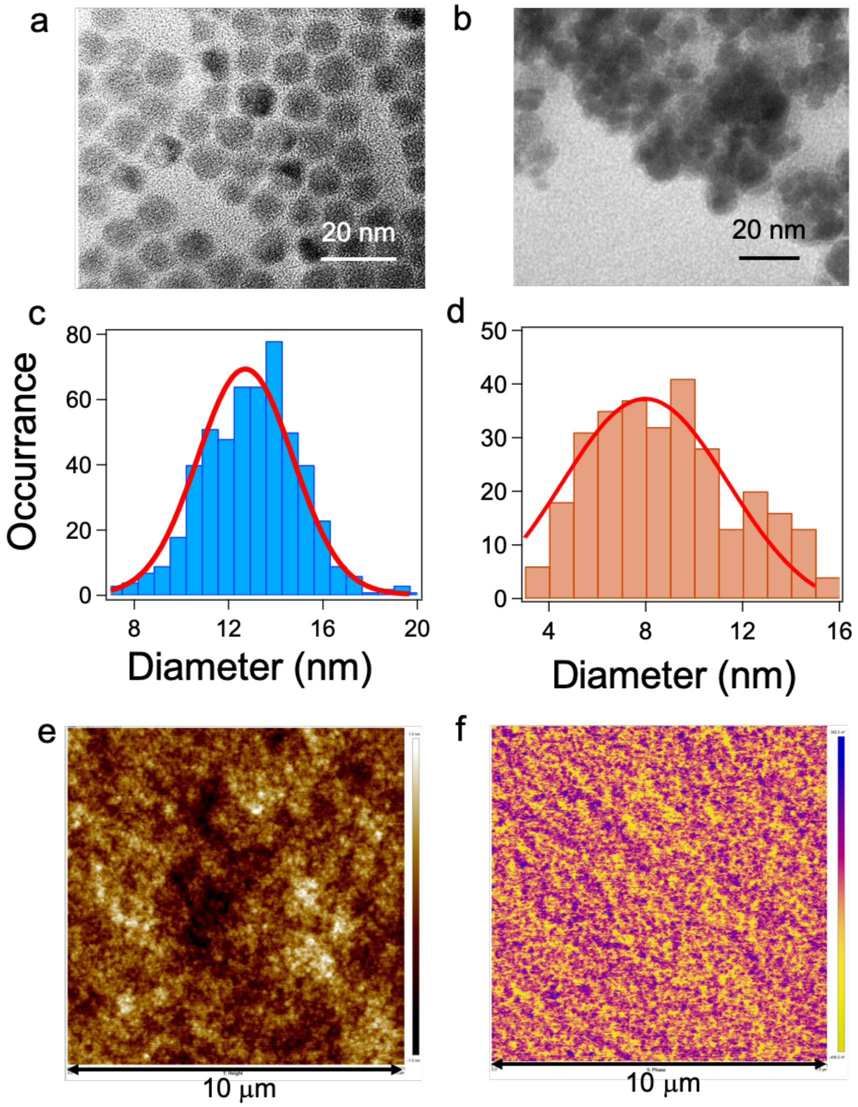

Figure S1. Transmission electron microscope (TEM) images of (a) the colloidal quantum dot (CdSe/CdS), and (b) iron oxide nanoparticle (Fe_3O_4) with (c,d) their corresponding size distribution histograms, respectively. (e) The 100 μm^2 morphological atomic force microscope (AFM) image of the 10-μm-thick hybrid material spin-coated on the glass substrate. (f) The magnetic force microscope (MFM) image of the corresponding film.

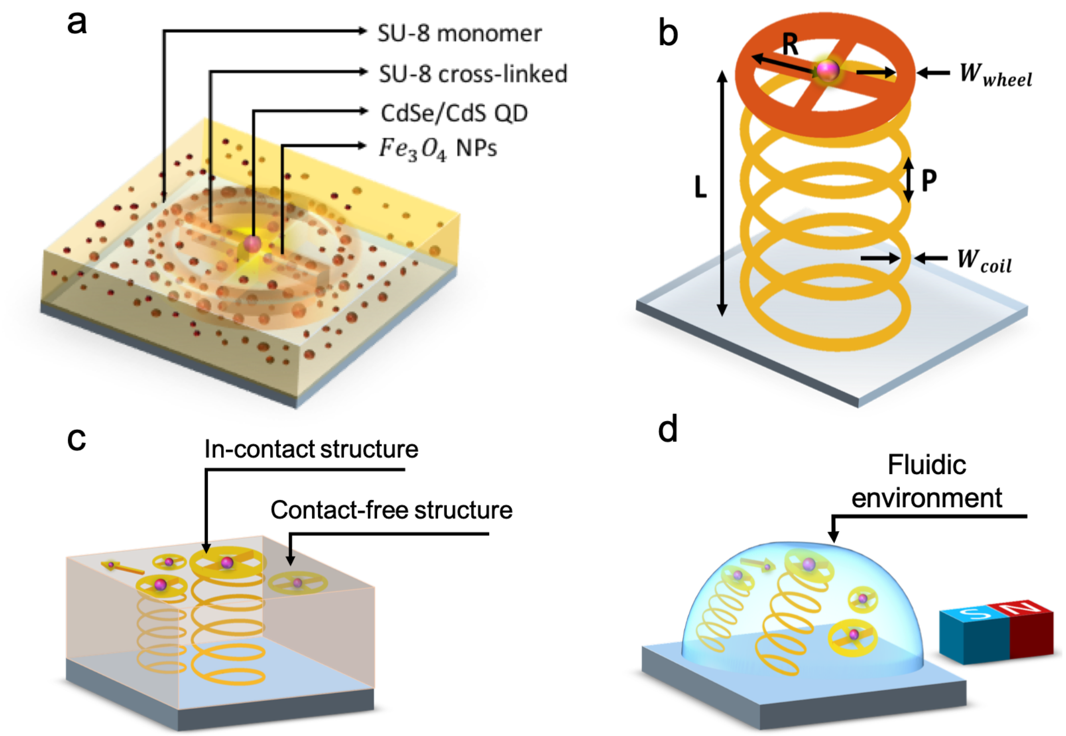

Figure S2. (a) Illustration of a contact-free device and chemical components within the structure. (b) Model illustration of in-contact device with its dimensions: radius, R = 3 μm, micro-wheel linewidth, W_{wheel} =1 μm, nano-coil linewidth, W_{coil} = 0.5 μm, vertical pitch, P = 1.5 μm, length of spring, L = 7 μm, and number of turns, N = 4, respectively. (c) Illustration of a sample containing both contact-free and in-contact devices right after the fabrication processes. (d) A bar magnet is introduced close to the sample in order to manipulate the structures in the fluidic environment.
